# Supplementary material for: RGB-Marking to Identify Patterns of Selection and Neutral Evolution in Human Osteosarcoma Models
Source: Cancers (Basel). 2021 Apr 21;13(9):2003. doi: 10.3390/cancers13092003 (PMC8122697; doi:10.3390/cancers13092003)
Supplement: Supplementary file 1 [file cancers-13-02003-s001.zip › cancers-1140951-supplementary.pdf]

Supplementary materials: RGB-marking to identify patterns of selection and neutral evolution in osteosarcoma models.

Stefano Gambera, Ana Patiño-García, Arantzazu Alfranca, Javier Garcia-Castro.

SUPPLEMENTARY FIGURES

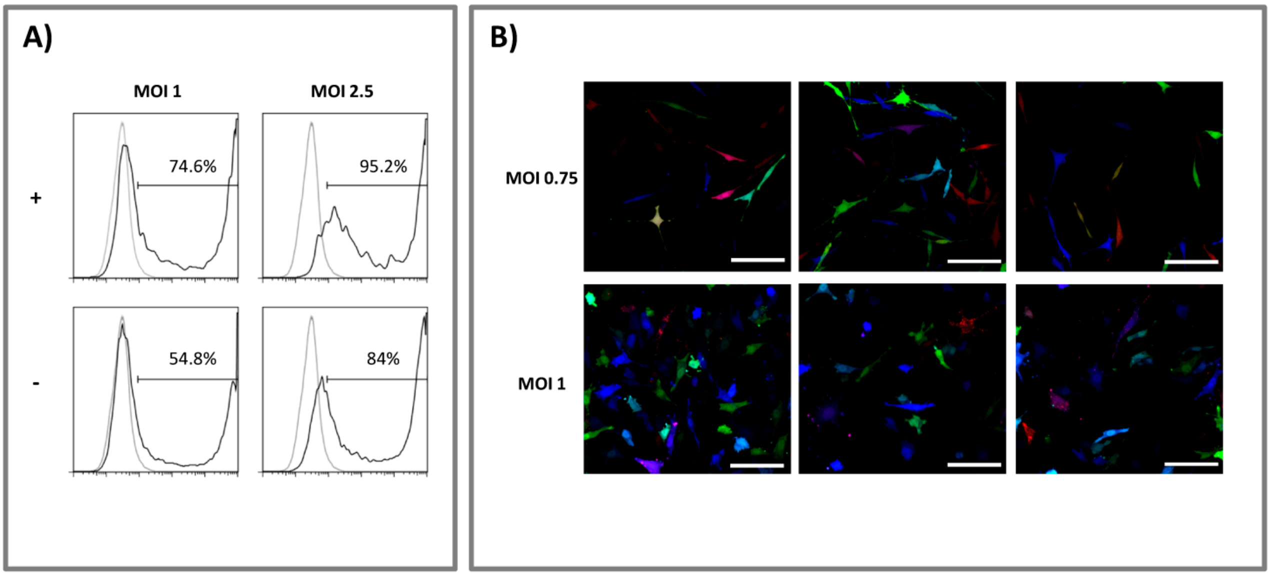

Figure S1: K5 OSA cells transduction and RGB marking validation.

The figure shows MOIs calculation and RGB marking validation process of K5 OSA cells. **A,** Fluorescent marker expression and optimal MOIs estimation. Polybrene was added to increase transduction efficiency but resulted in too high transduction efficiency. **B,** Representative confocal microscopy images of K5 RGB cells. Note that higher MOIs administration did not lead to increased color variability.

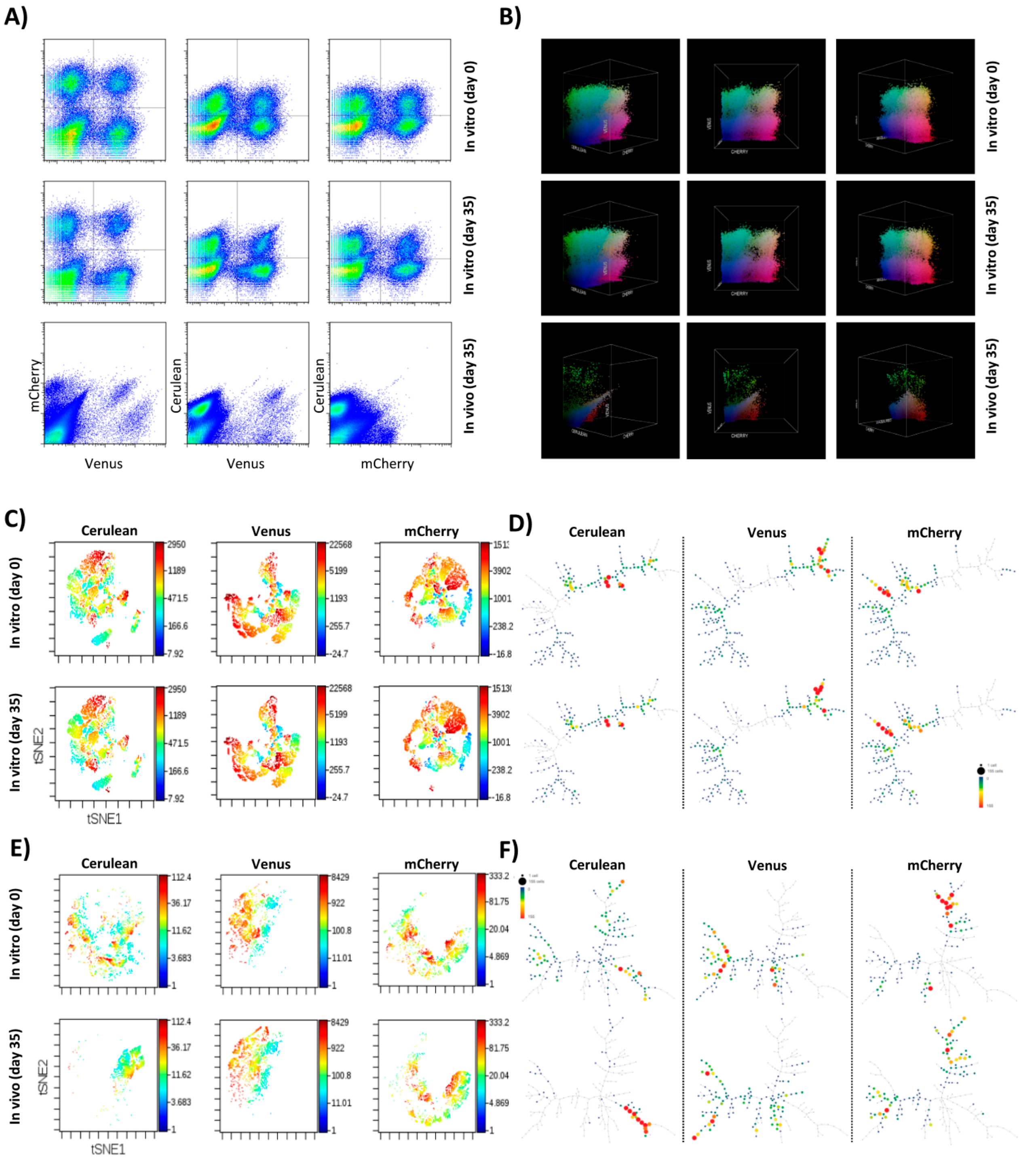

Figure S2: 3D flow cytometry, ViSNE and SPADE analysis represent useful tools for clonal studies based on LeGO-RGB system.

The figure shows the clonal composition of K5 RGB cells according to four different analyses methods. Fluorescent markers expression was acquired by flow cytometry. **A,** Clonal composition of K5 RGB cells at the starting point (top), 35 days after in vitro culture (middle) and 35 days after in vivo growth (bottom). Each

dot plot series correspond to a representative example of the 3 possible markers combinations obtained by analysing two fluorescent markers per time: Venus vs. mCherry, Venus vs. Cerulean and mCherry vs. Cerulean. Rainbow scale indicates cell frequency. **B,** Clonal composition of K5 RGB cells at starting point (top), 35 days after in vitro culture (middle) and 35 days after in vivo growth (bottom). Each 3D model represents the data presented in A, but visualizing simultaneously the three fluorescent variables (Cerulean, Venus and mCherry). Note the significant enrichment of some populations after in vivo growth. **C,** Representative viSNE analysis of K5 RGB cells at day 0 (top) vs day 35 (bottom) of in vitro culture. **D,** Representative SPADE analysis of K5 RGB cells in vitro at day 0 (top) vs day 35 (bottom). **E,** Representative viSNE analysis of K5 RGB cells in vitro (top) vs in vivo (bottom). **F,** Representative SPADE analysis of K5 RGB cells in vitro at day 0 (top) vs in vivo at day 35 (bottom).

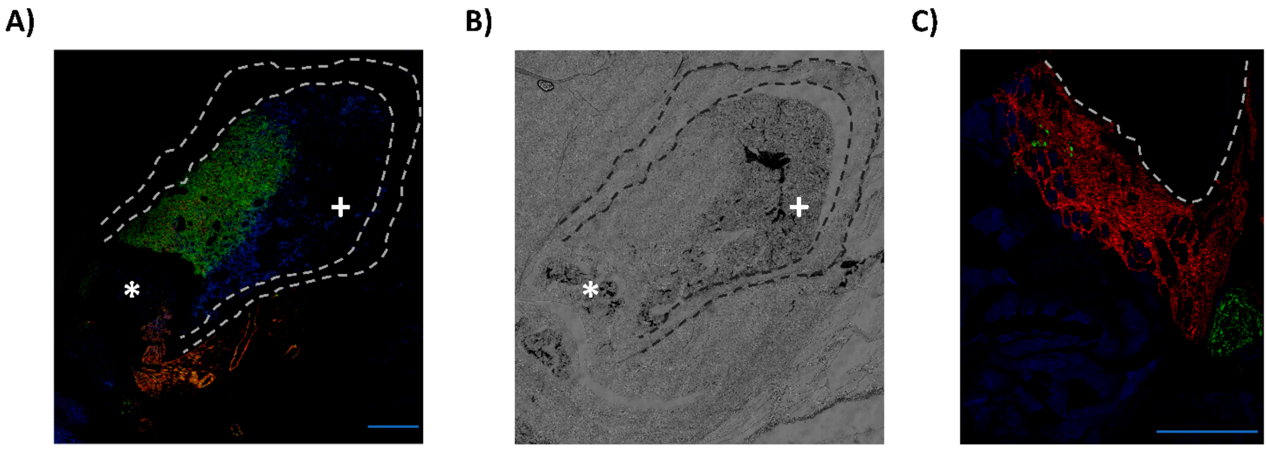

Figure S3: K5 RGB cells induce oligoclonal tumors at orthotopic location.

The figure shows the clonal composition of K5 RGB tumors induced at orthotopic location. **A,** Representative confocal microscopy tumor map showing a green clone developing intramedullary, and an orange clone invading the cartilaginous plate and overgrowing extramedullary. **B,** Phase contrast image of Figure S3A. **C,** Representative confocal microscopy image showing the infiltrative behavior of a bright red clone invading the surrounding muscular tissue. + indicates bone marrow, * indicates the epiphysis, and dotted line compact bone; blue bars=500 µm.

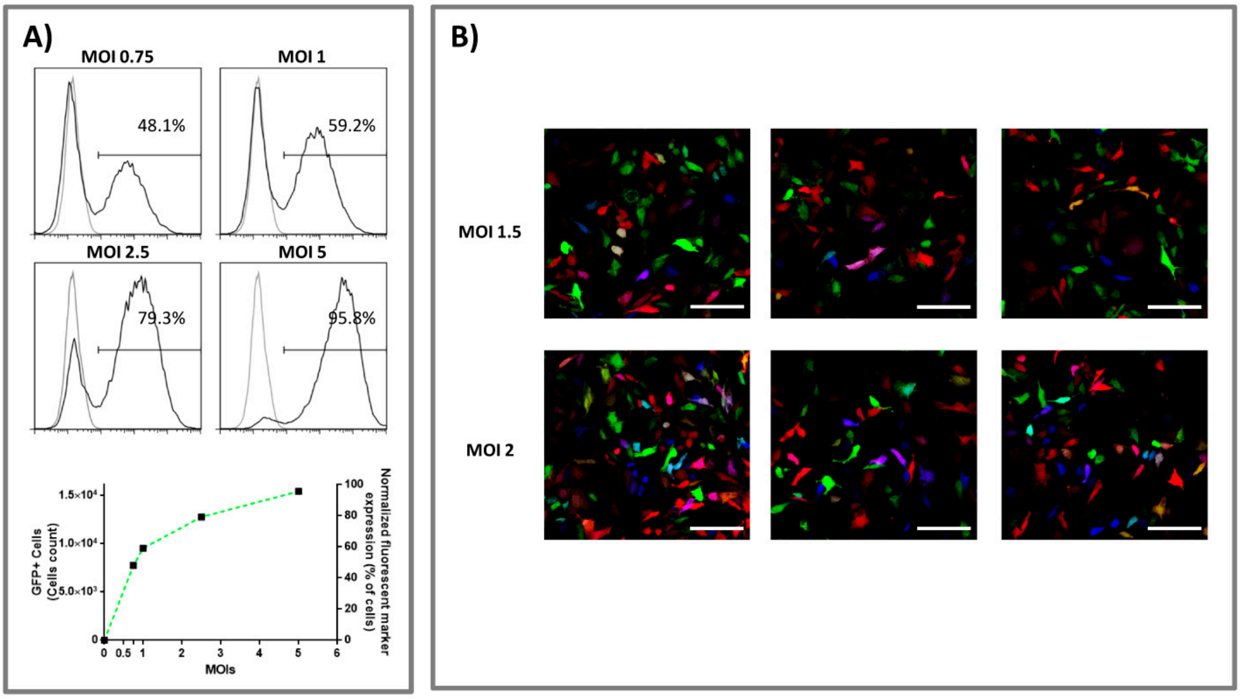

Figure S4: Saos2 cells transduction and RGB marking optimization.

The figure shows MOIs calculation and RGB marking validation process of Saos2 cells. **A,** Histograms (upper part of the panel) showing fluorescent marker expression of transduced Saos2 cells (black) vs un-transduced Saos2 cells (grey) at increasing vector concentration (MOIs). Percentages of transduction are reported and, in the lower part of the panel, an XY graph plot of the transduction efficiency. **B,** Representative confocal microscopy images of Saos2-RGB cells. Note that higher MOIs of cerulean vector administration generate more color variability.

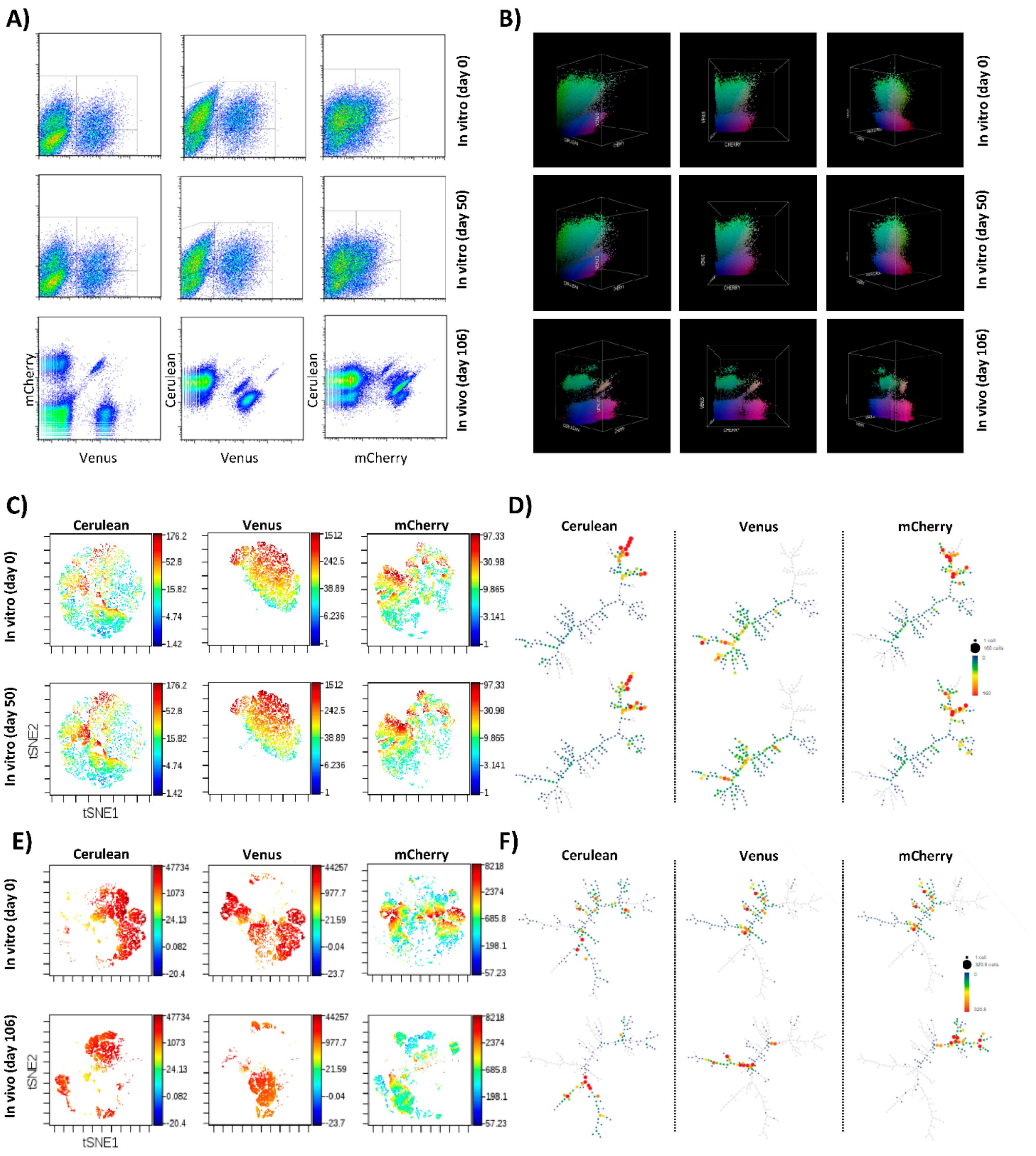

Figure S5: 3D flow cytometry, ViSNE and SPADE analysis indicate that Saos2 RGB cells undergo different clonal dynamics in vitro and in vivo.

The figure shows the clonal composition of Saos2-RGB cells after transduction (day 0), after in vitro culturing (50 days) and after in vivo growth (100 days). **A,** Clonal composition of Saos2-RGB cells; each dot plot series correspond to a representative example of the 3 possible markers combinations obtained analysing two fluorescent markers per time: Venus vs. mCherry, Venus vs. Cerulean and mCherry vs. Cerulean. Rainbow

scale indicates cell frequency. **B,** Clonal composition of Saos2-RGB cells; each 3D model represents the data presented in A, but visualizing simultaneously the three fluorescent variables (Cerulean, Venus and mCherry). Note the significant enrichment of some populations after in vivo growth. **C,** Representative viSNE analysis of K5 RGB cells at day 0 (top) vs day 50 (bottom) of in vitro culture. **D,** Representative SPADE analysis of Saos2-RGB cells in vitro at day 0 (top) vs day 50 (bottom); same data presented in C. **E,** Representative viSNE analysis of Saos2-RGB cells in vitro (top) vs in vivo (bottom). **F,** Representative SPADE analysis of Saos2-RGB cells in vitro at day 0 (top) vs in vivo at day 35 (bottom).

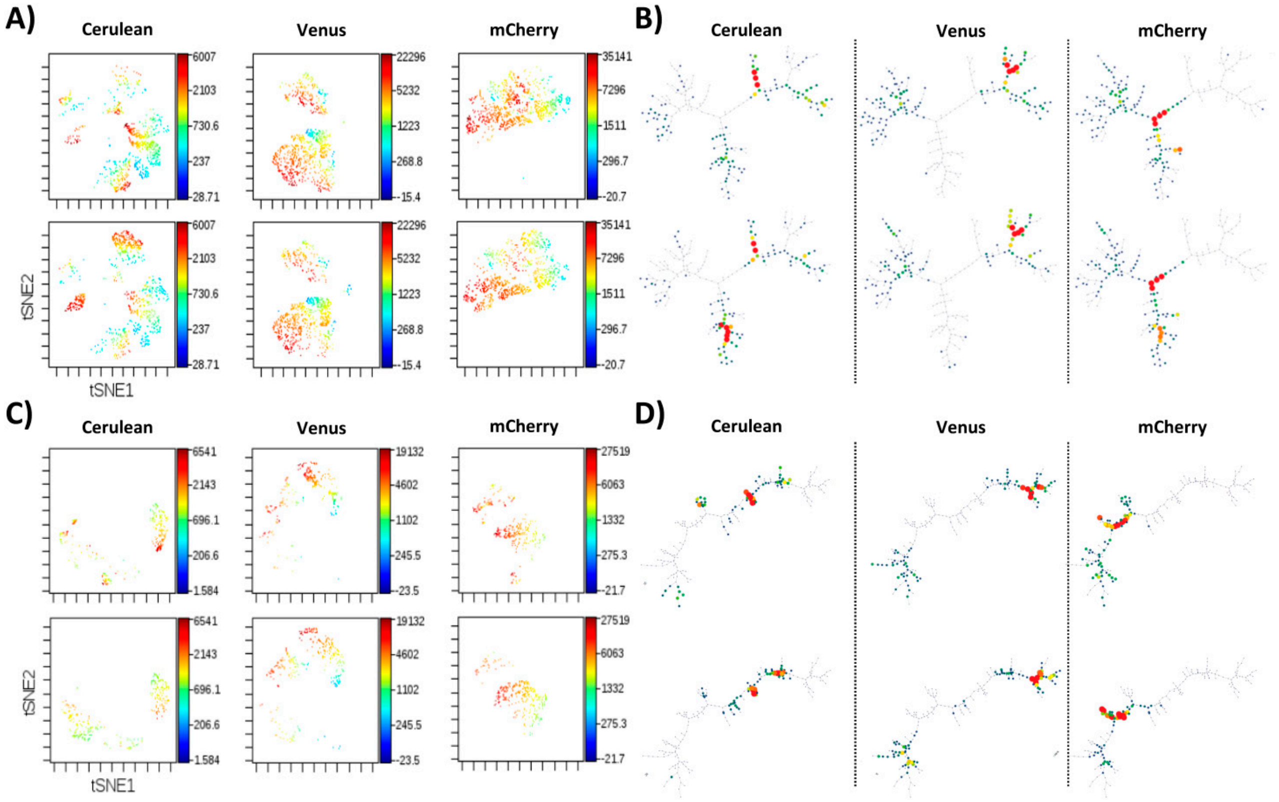

Figure S6: Tumor-derived clones are composed of sub-clones stably replicating in vitro and slightly changing in vivo.

The picture shows viSNE and SPADE analysis of re-traced tumor-derived clones at secondary transplantation. **A,** Representative viSNE analysis of re-traced clones at day 0 (top) and day 20 (bottom) of in vitro culture; corresponding SPADE analysis is presented in **B**. Rainbow scale indicates clonal frequency. **C,** Representative viSNE analysis of re-traced tumor-derived clones in vitro (top) and in vivo (bottom). Corresponding SPADE analysis is presented in **D**; rainbow scale indicates clonal frequency.

SUPPLEMENTARY TABLES

Table S1: RGB-marking K5 OSA cells and flow cytometry data comparison

FACSAria II

	Violet (405 nm)	Blue (488 nm)	Blue (488 nm)
Laser:	Violet (405 nm)	Blue (488 nm)	Blue (488 nm)
Filter:	AF-430 (525/50 nm)	FITC (530/30)	PE (585/15)
	**Cerulean +**	**Venus +**	**mCherry +**
K5 OSA Control	0.82	0.101	1.78
K5 OSA RGB (MOI 0.5)	**37**	30.4	**36.2**
K5 OSA RGB (MOI 1)	38.4	38.5	37.5

BD LSRFortesa X-20

Laser:	Violet (405 nm)	Blue (488 nm)	Yellow-Green (561 nm)
Filter:	BV-421 (450/50 nm)	FITC (530/30 nm)	PE-Texas red (610/20 nm)
	**Cerulean +**	**Venus +**	**mCherry +**
K5 OSA	1.00	0.201	0.809
K5 OSA RGB (MOI 0.5)	**34.3**	27.2	**27.9**

Fluorescent markers quantification of K5 OSA cells at different MOIs and the same cells (MOI 0.5) according to two different flow cytometers and filter/laser settings. Note that some hardware settings can introduce cell frequency estimation bias and require a more appropriate set of Laser/Filter.

Table S2: RGB-marking Saos2 cells

FACSAria II

Laser:	Violet (405 nm)	Blue (488 nm)	Blue (488 nm)
Filter:	AF-430 (525/50 nm)	FITC (530/30)	PE-Texas Red ()
	**Cerulean +**	**Venus + (MOI 1)**	**mCherry + (MOI 2)**
Saos2 MIX D (MOI 2)	80.4	26	38.2
Saos2 MIX C (MOI 1.5)	71.6	26	35.5
Saos2 MIX B (MOI 1)	38.2	24.2	38.1
Saos2 MIX A (MOI 0.5)	15.5	26.6	38.6

Fluorescent markers quantification of Saos2-RGB cells at increasing cerulean vector administration. Note that Saos2-RGB cells required an 80% of transduction to obtain an appropriate full spectrum of color combinations

generated by an optimal fluorescent proteins mix (see Figure S5B); Venus and mCherry expression in around 30% of cells was sufficient for an efficient RGB-marking.
